# The m6A Reader YTHDF3 Promotes Glioma Progression by Regulating the m6A Modification of lncRNA‐PAR5

**DOI:** 10.1155/ancp/8467384

**Published:** 2026-04-16

**Authors:** LiuFei Xu, Yuan Xu, YongBin Duan, Bo Wang, YiYu Luo, XiangPeng Wang

**Affiliations:** ^1^ Department of Neurosurgery, First Affiliated Hospital of Kunming Medical University, Kunming, 650000, Yunnan, China, kmmc.cn

**Keywords:** glioma, invasion, lncRNA-PAR5, m6A modification, migration, proliferation, YTHDF3

## Abstract

**Background:**

Long non‐coding RNA PAR5 (lncRNA‐PAR5) is downregulated in glioma and has been confirmed to inhibit glioma progression; however, the specific regulatory mechanism underlying its downregulation remains unclear.

**Objective:**

This study aimed to investigate the key molecular mechanism by which PAR5 inhibits glioma progression, with a focus on the regulatory role of m6A modification.

**Methods:**

Potential m6A modification sites in the PAR5 sequence were predicted using the SRAMP online tool. The expression profiles of m6A regulatory genes in glioblastoma were analyzed via the GEPIA database. RNA pull‐down, RIP‐qPCR, and MeRIP‐qPCR were employed to validate the specific binding of YTHDF3 to PAR5 and its effect on the m6A modification level of PAR5. The expression of PAR5 and YTHDF3 was modulated by cell transfection, and cell proliferation, invasion, and migration were assessed using CCK‐8, Transwell, and wound healing assays, respectively. Further in vivo functional validation was performed using a subcutaneous xenograft tumor model in nude mice.

**Results:**

lncRNA‐PAR5 significantly inhibited the proliferation, invasion, and migration of glioma cells. Bioinformatics analysis and experimental validation revealed that the m6A reader protein YTHDF3 is highly expressed in glioma, specifically recognizes and binds to PAR5, and promotes PAR5 degradation by enhancing its m6A modification level, thereby negatively regulating PAR5 expression. Functional experiments demonstrated that YTHDF3 plays a pro‑oncogenic role, while knockdown of YTHDF3 suppressed malignant phenotypes of glioma, an effect that could be partially reversed by simultaneous knockdown of PAR5. In vivo experiments further confirmed that YTHDF3 knockdown inhibits tumor growth by upregulating PAR5.

**Conclusion:**

YTHDF3 promotes glioma cell proliferation, invasion, and migration by inhibiting PAR5 expression through enhancing its m6A modification. This study reveals the critical role of the YTHDF3/PAR5 axis in glioma progression and provides a potential novel target for glioma‑targeted therapy.

## 1. Introduction

Glioma is a common type of brain tumor originating from glial cells, with high heterogeneity and invasive proliferation [1]. Surgery, chemotherapy, and radiotherapy are conventional therapies for glioma [2]. Over the past few decades, although scientists have made some progress in clinical and basic research as well as comprehensive treatment, the mortality rate of glioma remains inadequately controlled due to its high rates of metastasis and recurrence [3]. Several factors influence the progression of glioma and the survival of patients, including tumor location, patient age, cancer cell mutation, and epigenetic disorders [4, 5]. Therefore, it is urgent to further study the mechanism of the occurrence and development of glioma and develop new treatment strategies.

Long non‐coding RNA (lncRNA) is a non‐protein‐coding transcript that comprises of >200 nucleotides. It is often found to be dysregulated in a variety of cancers, including glioma, and can be used as a biomarker and therapeutic target to provide patients with more effective treatment, diagnosis, and prognosis [6–8]. More and more lncRNAs are involved in the biological and pathological processes of glioma, such as metabolic reprograming, proliferation, invasion, apoptosis, radiotherapy, and chemotherapy [9, 10]. lncRNA Prader Willi/Angelman region RNA5 (PAR5) has been proposed as a new tumor suppressor lncRNA [7]. Our previous studies have confirmed that PAR5 is significantly down‐regulated in glioma tissues and cell lines. Patients with low expression of PAR5 have poor overall survival. Restoration of PAR5 expression in vitro inhibits the proliferation, invasion, and migration of human glioma cells by binding to EZH2 and regulating oncogene expression [11]. However, it is not clear what causes the decrease of PAR5 expression in glioma.

In a variety of complex diseases, the expression level and function of lncRNA are significantly affected by genomic methylation [9]. It has been reported that N6‐methyladenosine (m6A) modification is the most abundant RNA modification, which involves the methylation of the sixth nitrogen atom of the adenine (A) molecule in messenger RNA (mRNA) or non‐coding RNA (ncRNA) [12]. M6A methylation affects almost all aspects of RNA metabolism, including RNA translocation, splicing, stabilization, and translation [13]. The m6A modification is regulated by “writer” proteins (METTL3, METTL14, etc.), “eraser” proteins (FTO, ALKBH5, etc.), “reader” proteins (YTHDC13, YTHDF1‐3, etc.) [14]. Among these, YTHDF3 has been found to play a critical role in multiple cancer types. For instance, it promotes breast cancer brain metastasis by enhancing the interaction between cancer cells and brain endothelial cells and astrocytes, increasing blood–brain barrier permeability, and promoting angiogenesis and tumor growth [15]. In hepatocellular carcinoma, higher expression of YTHDF3 is associated with poor prognosis [16] and increased tumor invasiveness [17]. Additionally, YTHDF3 mediates the regulation of HNF1α on radioresistance in cervical cancer by promoting RAD51D translation in an m6A‐dependent manner [18]. In clear cell renal carcinoma, YTHDF3 enhances immune evasion by phase separation and recruiting DDX6 to promote the degradation of its target mRNA HSPA13 [19]. In glioma, m6A modification also plays a central role in tumor progression. Studies have shown that m6A regulatory factors promote tumor cell proliferation [20] and invasion [21], contribute to resistance to therapies—particularly to temozolomide (TMZ) [22]—and support the maintenance and self‐renewal of glioblastoma stem‐like cells [23]. Key m6A regulators such as ALKBH5, YTHDC1, FTO, and METTL3 not only participate in tumor cell growth but also influence the immune microenvironment [24]. Additionally, the m6A score has prognostic significance, aiding in predicting patient outcomes and informing clinical decision‐making [25]. Recent studies have indicated that PAR5 is a lncRNA associated with m6A [26], and a potential m6A modification motif in the PAR5 sequence was also found by the SRAMP prediction server. Therefore, we speculate that the decrease of PAR5 expression in glioma may be related to m6A. This study aims to systematically investigate the regulatory mechanism of m6A modification on PAR5 expression, providing new theoretical insights into lncRNA epigenetic regulation in glioma and laying the groundwork for potential biomarkers and therapeutic targets.

In this paper, seven common m6A regulatory factors (METTL3, METTL14, YTHDF1, YTHDF2, YTHDF3, FTO, and ALKBH5) were found according to the literature [24, 27], and the genes abnormally expressed in glioma were found in GEPIA. RNA pulldown was used to detect m6A regulatory genes that bind to lncRNA‐PAR5, and verify whether this gene is a key gene that mediates abnormal PAR5 expression.

## 2. Materials and Methods

### 2.1. Cell Culture

All cells were obtained from the Cell Bank of the Chinese Academy of Sciences (Shanghai, China). U87, SHG44, U251, and T98 cells were cultured in a medium containing 10% fetal bovine serum (FBS), with a passaging ratio of 1:2, and the medium was changed twice a week. After 3 days of cultivation, when the cell confluence reached 75%, the cells were passaged and exhibited adherent growth. Human skin fibroblasts (HF) were cultured in medium containing 15% FBS, with a passaging ratio of 1:2 and medium was changed three times a week. After 5 days of cultivation, when the cell confluence reached 80%, the cells were passaged and exhibited adherent growth. Human oligodendrocyte cell lines (OL) were cultured in medium containing 20% FBS, with a passaging ratio of 1:4 and medium was changed twice a week. After 5 days of cultivation, when the cell confluence reached 80%, the cells were passaged and exhibited adherent growth. All cells were cultured in Dulbecco’s Modified Eagle Medium (DMEM, Gibco, USA) containing 100 U/mL penicillin and 100 μg/mL streptomycin (BioWest, France), in a 37°C incubator with 5% CO_2_ and saturated humidity. Before use in experiments, all cells were tested for HIV‐1, HBV, HCV, mycoplasma, bacteria, yeast, and fungi and were negative.

### 2.2. Cell Transfection

Small interfering RNAs (si‐PAR5, si‐YTHDF3) and overexpressing the vector plasmid pc‐YTHDF3 specifically targeting human PAR5 and YTHDF3 mRNA were purchased from RiboBio (Guangzhou, China). According to the manufacturer’s protocol, U87 cells with a final concentration of 2 × 10^5^ were seeded into each well of a 6‐well plate. One hour before transfection, the medium was replaced with fresh serum‐free, antibiotic‐free medium. The DNA‐Lipo2000 (Invitrogen, USA) complexes were prepared at a DNA: Lipo2000 ratio of 1:2 and added to the cell culture medium. The plate was gently shaken to ensure even distribution of the complexes. Afterward, the plate was incubated at 37°C with 5% CO_2_ for 48 h, with the medium being changed at 6 h post‐transfection. Following transfection, the mRNA levels of the target gene PAR5 were assessed by qRT‐PCR, while the protein levels of the target gene YTHDF3 were determined by Western blot to verify transfection efficiency.

### 2.3. CCK‐8

Cells (2 × 10^3^ cells/well) from each group were added to a 96‐well plate and cultured in an incubator at 37°C for 24 h. Then 10 μL CCK‐8 solution (cat: C0038, Beyotime, China) was added. Incubation was continued in an incubator at 37°C for 30 min, and the absorbance was detected at a wavelength of 450 nm using a microplate reader.

### 2.4. Transwell

The cells were inoculated into Transwell chamber coated with Matrigel (cat: FTW001‐24lns, Beyotime, China). 500 μL of medium containing FBS was added to the lower chamber. After culturing for 24 h at 37°C, 5% CO_2_, and 90% humidity, the Transwell chamber was removed and the culture medium, matrix gel, and cells inside the chamber were removed. The lower surface was immersed in 4% paraformaldehyde for 10 min. After washing, the samples were stained with 10% crystal violet (cat: 60505ES25, YEASEN, China) for 5–10 min. Wash with PBS to remove crystal violet that is not bound to the cell. Cells that did not penetrate the Transwell chamber membrane were removed from the upper layer with cotton swabs. After air‐drying, the cell invasion was observed under a microscope, and ImageJ was used to count.

### 2.5. Wound Healing

Cells were inoculated into a 12‐well plate. When cell confluence reached ~90%, a straight scratch was created on the monolayer using a sterile 200 µL pipette tip to simulate a “wound.” The scratch healing process was observed and recorded under a microscope at 0 and 24 h after wounding. The cell migration rate was subsequently analyzed using ImageJ software.

### 2.6. RT‐qPCR

According to the manufacturer’s instructions, use TRIzol assay kit (cat: 12183555, Invitrogen, USA) to extract total RNA. A one‐step PrimeScript cDNA synthesis kit (cat: BTN130609, Beijing Baiaolaibo Technology Co., Ltd, China) was used to reverse transcribe RNA into cDNA. The SYBR Green PCR (cat: BTN90408, Beijing Baiaolaibo Technology Co., Ltd, China) premix was used for PCR analysis in a 7900HT real‐time fluorescent quantitative PCR system (Life Technologies, USA). Relative expression analysis was conducted by applying the 2^−∆∆CT^ method, with GAPDH as the internal reference. lncRNA‐PAR5 Forward: GATCTTGGCTCACCGCAACCTC, Reverse: CAACCTGGCAAACATGGCAAAGC.

### 2.7. RNA Pulldown

Cells were collected, total RNA was extracted and RNA fragmentation was performed. Biotin RNA probes were transcribed in vitro using a biotin RNA labeling mixture (cat: 11277073910, Roche Diagnostics) and T7 RNA polymerase (cat: RPOLT7‐RO, Roche Diagnostics). According to the instructions of RNA pull‐down positive kit (cat: KT103, Guangzhou Saicheng Biotechnology Co., Ltd, China), RNA probes were combined with streptavidin‐labeled magnetic beads to form RNA‐magnetic bead complexes. The RNA‐magnetic bead probe was incubated with the cell lysate at 4°C for one night, and the magnetic beads were washed. Loading buffer was added and boiled at 100°C for 10 min, and centrifuged at 3000 rpm for 1 min. The supernatant was the RNA pull‐down product. The expression of METTL14, YTHDF3, and FTO in PAR5–protein complex was analyzed by Western blot.

### 2.8. MeRIP‐qPCR

Cells in each group were collected, total RNA was extracted and RNA fragmentation was performed. 2 μL RNA sample was used as Input. According to the kit instructions, total RNA was used for m6A‐IP using EpiMark N6‐Methyladenosine Enrichment Kit (cat: E1610S, NEB, USA). RT‐qPCR detected the expression of lncRNA‐PAR5 in IP solution.

### 2.9. RIP‐qPCR

The cells were washed twice with PBS and centrifuged at 1500 rpm for 5 min at 4°C. Cells were lysed using RIP lysis buffer. After incubating on ice for 5 min, the lysate was harvested by centrifugation at 12,000 rpm for 10 min. RIP analysis was performed using Magna RIP Kit (cat: 17‐700, Millipore, Germany) according to manufacturer’s instructions. The magnetic beads were mixed with 5 μg anti‐YTHDF3 (cat: ab220161, Abcam, UK) or anti‐rabbit IgG (cat: ab6721, Abcam, UK) and incubated at 4°C for 4 h to prepare the magnetic bead–antibody complex. After washing with RIP Wash Buffer, the magnetic bead–antibody complex was incubated with cell lysates at 4°C overnight. The beads were washed three times with RIP Wash Buffer containing RNase inhibitors, and the co‐precipitated RNA was eluted from the immunoprecipitated complex. RT‐qPCR was used to detect the enrichment of lncRNA‐PAR5 in co‐precipitated RNA.

### 2.10. RNA Stability Assays

U87 cells in the logarithmic growth phase were seeded at a density of 6 × 10^3^ cells per well in a 24‐well plate and cultured in high‐glucose DMEM medium. After 24 h, actinomycin D was added to each well at a final concentration of 5 μg/mL. Cells were collected before drug addition (0 h) and at 2, 4, and 6 h after treatment. Total RNA was extracted using TRIzol reagent, and the expression level of lncRNA‐PAR5 was measured by RT‑qPCR to assess its stability.

### 2.11. Western Blot

Total protein was isolated from cells using a protein extraction kit and the protein concentration was determined using a BCA assay kit (cat: P0009, Beyotime, China). The protein was loaded into the electrophoresis chamber of sodium dodecyl sulfate‐polyacrylamide gel electrophoresis (SDS‐PAGE), and then transferred to the nitrocellulose membrane. The membrane was blocked with 5% skim milk at room temperature for 1 h. After that, the PVDF membrane was treated with primary antibody (YTHDF3 (cat: A8395, ABclonal, China): 1:1000, GAPDH (cat: A19056, ABclonal, China): 1:2000) at 4°C overnight, and then incubated with the corresponding secondary antibody (cat: AS009, ABclonal, China) for 1 h. ECL kit (cat: BL520A, Biosharp, China) was used for chemiluminescence detection and Image J was used for normalization analysis.

### 2.12. Tumor Xenograft Animal Experiments

The animal experimental protocols in this study were approved by the The Animal Experiment Ethics Review Committee of Kunming Medical University (kmmu20230611). Four‐week‐old male Balb/c nude mice (*n* = 6) purchased from Beijing Huafukang Biotechnology Co., Ltd. (China) were housed under specific pathogen‐free (SPF) conditions. Subsequently, U87 cells (1 × 10^7^ cells) subjected to different treatments were subcutaneously injected into the nude mice. The treatment groups included: transfection with negative control siRNA (si‐NC), knockdown of YTHDF3 alone (si‐YTHDF3), and simultaneous knockdown of YTHDF3 and lncRNA PAR5 (si‐YTHDF3 + si‐PAR5). During the experiment, the mice were housed at a density of five per cage with free access to food and water. On day 24 post‐inoculation, the mice were euthanized, and tumor tissues were completely excised. After photographic documentation, the tumors were weighed for subsequent analysis. Western blotting (WB) was performed to detect the protein expression level of YTHDF3, while real‐time quantitative PCR (RT‐qPCR) was conducted to measure the transcriptional level of lncRNA PAR5 in the tumor tissues.

### 2.13. Statistical Analysis

The SPSS software, version 26.0 (SPSS, Inc., Chicago, IL, USA) and GraphPad Prism 8.0 (GraphPad Software) were used for statistical analysis. Data are presented as the mean ± standard deviation. The nonparametric unpaired *T* test was utilized to calculate the *p* value of the difference between two independent datasets. One‐way analysis of variance (ANOVA) was used to analyze the significance among three or more independent datasets, and post‐hoc Tukey’s test was used to assess significant differences. The significance level was set at a *p*‐value less than 0.05, with differences considered statistically significant if *p*  < 0.05. All experiments were repeated three times.

## 3. Results

### 3.1. lncRNA‐PAR5 Inhibits the Proliferation, Invasion, and Migration of Glioma Cells

Our previous studies have confirmed that PAR5 is significantly down‐regulated in glioma tissues and cell lines [11]. In this experiment, we also found that the expression of PAR5 was decreased in glioma cells, and the expression was the lowest in U87 cells (Figure 1A). Furthermore, the effects of PAR5 on the proliferation, invasion, and migration of human glioma cells were verified at the cellular level by knocking down and overexpressing PAR5. The efficiency of PAR5 knockdown and overexpression is shown in Figure 1B. Overexpression of PAR5 significantly inhibited the proliferation, invasion, and migration of human glioma cells, while knockdown of PAR5 resulted in the opposite results (Figure 1C–E). This indicates that PAR5 plays a tumor suppressor role in glioma.

Figure 1lncRNA‐PAR5 inhibits the proliferation, invasion, and migration of glioma cells. (A and B) PAR5 expression was detected by RT‐qPCR; (C) CCK‐8 assay was used to detect cell proliferation activity; (D) Transwell was used to detect cell invasion ability. Scale bar = 50 μm; (E) Wound healing assay was used to detect cell migration ability. Scale bar = 100 μm Compared with negative control (NC),  ^∗^ *p*  < 0.05,  ^∗∗^ *p*  < 0.01,  ^∗∗∗^ *p*  < 0.001.

**Figure:**
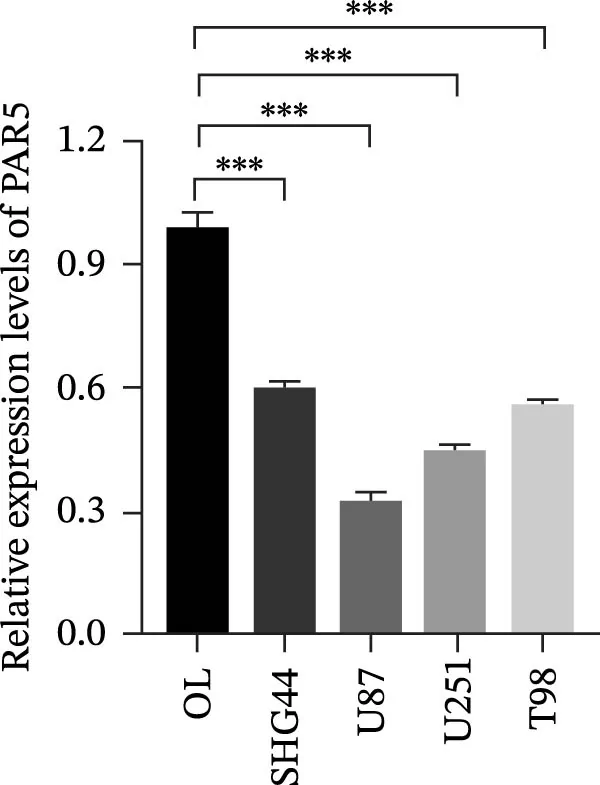
(A)

**Figure:**
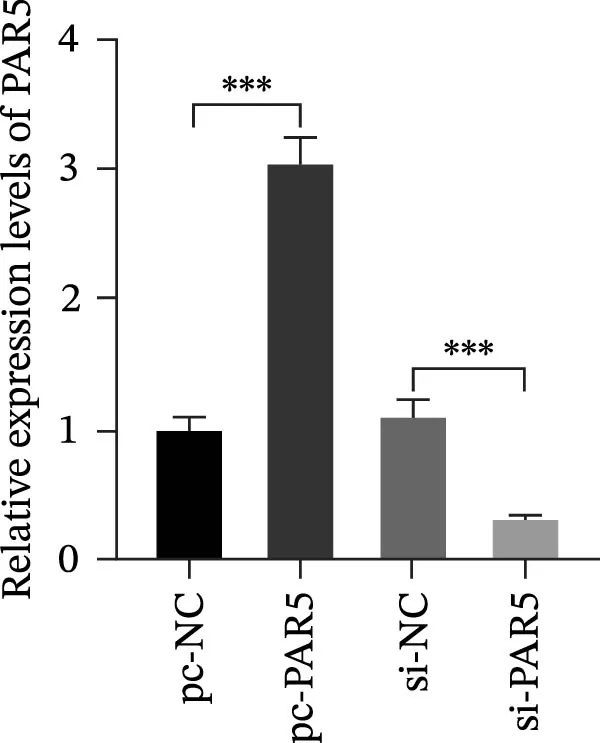
(B)

**Figure:**
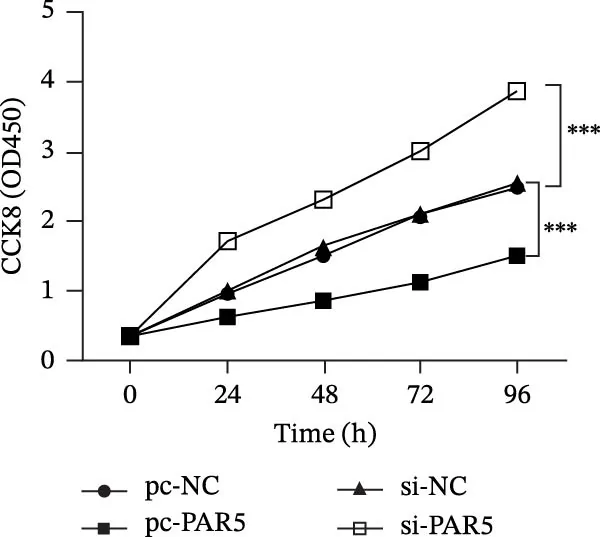
(C)

**Figure:**
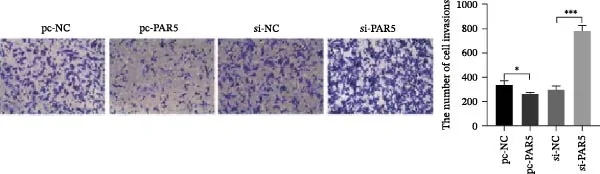
(D)

**Figure:**
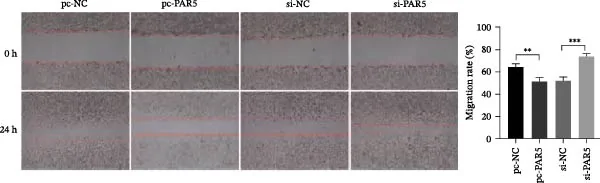
(E)

### 3.2. The Expression of lncRNA‐PAR5 May be Regulated by m6A Modification

Since lncRNA expression is easily affected by m6A modification [28], we used the SRAMP prediction server to analyze whether PAR5 contains m6A modification sites. The results showed that within the 0–500 nucleotide region, a predicted m6A site “GGAU” was identified with a combined score falling into the moderate confidence range (Figure 2A), suggesting it may be subject to m6A regulation. To further explore the potential role of m6A modification in the regulation of PAR5 in glioblastoma, we first examined the expression of m6A regulatory factors in glioblastoma. Using the GEPIA database, we analyzed common m6A‐related genes including METTL3, METTL14, YTHDF1, YTHDF2, YTHDF3, FTO, and ALKBH5. The results indicated that METTL14, YTHDF1, YTHDF2, and YTHDF3 were significantly upregulated in GBM. (Figure 2B). RT‐qPCR validation further confirmed that METTL14, YTHDF3, YTHDF1, and YTHDF2 were markedly overexpressed in glioma cells. (Figure 2C). To determine whether these m6A regulators specifically interact with PAR5 in glioma cells, we conducted RNA pull‐down assays. The results demonstrated that only YTHDF3 specifically binds to PAR5 (Figure 2D). It can be seen that the expression of PAR5 may be regulated by m6A modification.

Figure 2The expression of lncRNA‐PAR5 may be regulated by m6A modification. (A) SRAMP prediction server (https://www.cuilab.cn/sramp) predicts the potential motif of m6A modification in PAR5 sequence. (B) Find the abnormal expression of m6A regulatory genes in GBM at GEPIA. (C) RT‐qPCR was used to detect the expression of METTL14, and YTHDF3 in glioma cells. (D) RNA pulldown was used to detect whether the YTHDF3 and METTL14 specifically bind to PAR5. Compared with OL cells,  ^∗^ *p*  < 0.05,  ^∗∗∗^ *p*  < 0.001.

**Figure:**
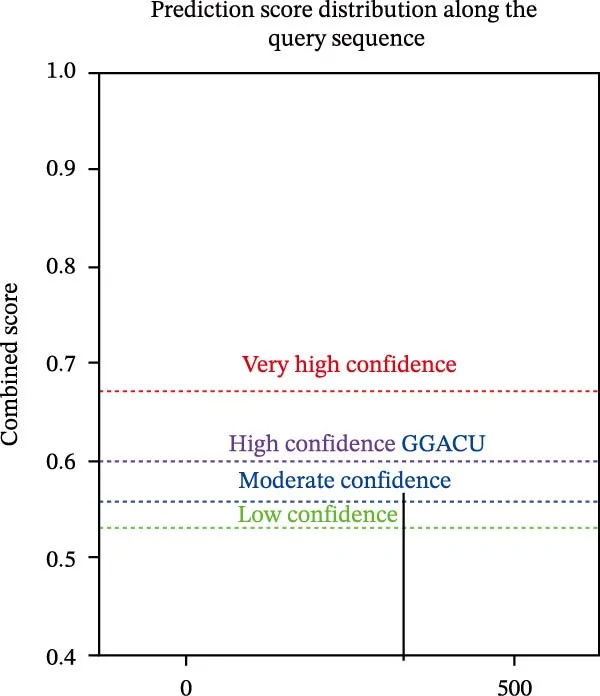
(A)

**Figure:**
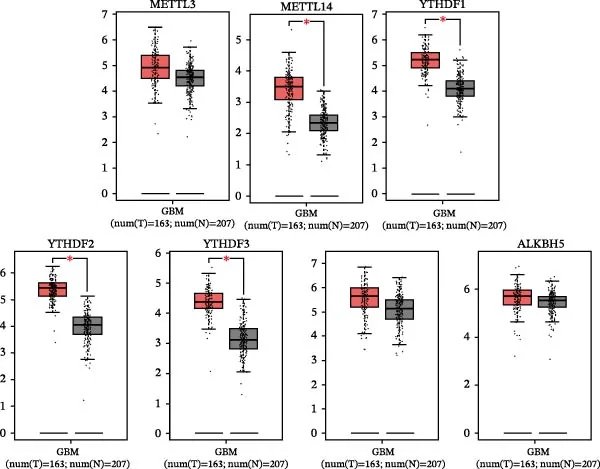
(B)

**Figure:**
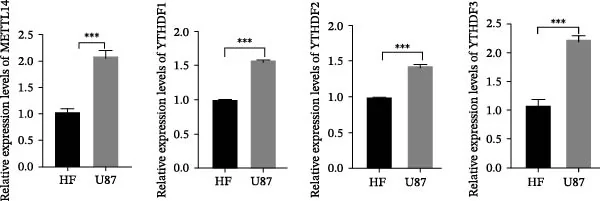
(C)

**Figure:**
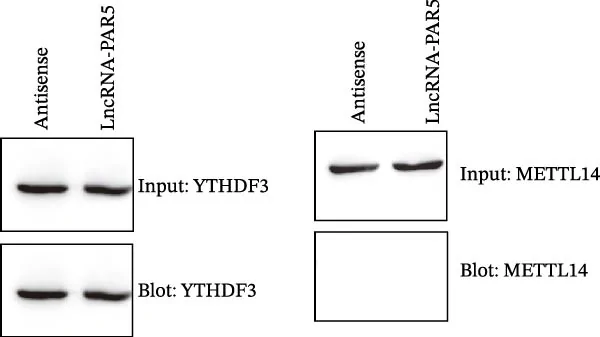
(D)

### 3.3. YTHDF3‐Mediated m6A Modification Inhibits PAR5 Expression

To validate the inhibitory effect of YTHDF3‐mediated m6A modification on PAR5 expression, we performed Western blot analysis to examine YTHDF3 expression in various glioma cell lines and normal OL cells. The results showed a significant increase in YTHDF3 expression in glioma cell lines (Figure 3A). Additionally, Figure 1A indicated a decrease in PAR5 expression in glioma cells, suggesting a potential negative regulatory relationship between YTHDF3 and PAR5. To further confirm this negative regulation, we first analyzed the correlation between YTHDF3 and lncRNA‐PAR5 in gliomas using ChIPBase [29]. The results revealed a significant negative correlation between YTHDF3 and PAR5 expression, with a *p*‐value less than 0.05 (Figure 3B). Next, through YTHDF3 knockdown and overexpression experiments, we verified this negative correlation: knockdown of YTHDF3 significantly upregulated PAR5 expression, while overexpression of YTHDF3 significantly downregulated PAR5 expression (Figure 3D). The efficiency of YTHDF3 knockdown and overexpression is shown in Figure 3C. The m6A modification of lncRNA‐PAR5 was detected by MeRIP‐qPCR. The results showed that knockdown of YTHDF3 significantly inhibited the m6A modification of lncRNA‐PAR5, and overexpression of YTHDF3 significantly promoted the m6A modification of lncRNA‐PAR5 (Figure 3E). RIP‐qPCR further confirmed the interaction between YTHDF3 and lncRNA‐PAR5 in glioma cells. The results showed that lncRNA‐PAR5 was apparently enriched by YTHDF3 immunoprecipitation (Figure 3F). To further investigate whether YTHDF3 regulates the expression of lncRNA‐PAR5 by affecting its stability, we measured the half‐life of lncRNA‐PAR5 in different groups of cells using the transcriptional inhibitor actinomycin D. The results showed that knockdown of YTHDF3 prolonged the half‐life of lncRNA‐PAR5 (Figure 3G). These findings indicate that YTHDF3 negatively regulates the expression stability of lncRNA‐PAR5 through m6A modification.

Figure 3YTHDF3‐mediated m6A modification inhibits PAR5 expression. (A and C) Western blot was used to detect YTHDF3 expression. (B) ChIPBase was used to analyze the co‐expression patterns of YTHDF3, and lncRNA PAR5 in glioblastoma, with blue dots representing tumor samples. *Y*‐axis: Expression level of PAR5 (FPKM), with a starting point of 0 and intervals of 0.25. *X*‐axis: Expression level of YTHDF3 (FPKM), with a starting point of 5 and intervals of 2.5. Regression line: *y* = −0.0087*x* + 0.4131. Correlation: YTHDF3 vs. PAR5 (*r* = 0.4997, *p*‐value = 1.48e–78), *n* = 342. (D) The expression of PAR5 was detected by RT‐qPCR. (E) MeRIP‐qPCR was used to detect the m6A modification of PAR5. (F) RIP‐qPCR confirmed the interaction between YTHDF3 and PAR5 in glioma cells. (G) Half‐life analysis of lncRNA‐PAR5 following YTHDF3 knockdown. Compared with NC,  ^∗^ *p*  < 0.05,  ^∗∗^ *p*  < 0.01,  ^∗∗∗^ *p*  < 0.001.

**Figure:**
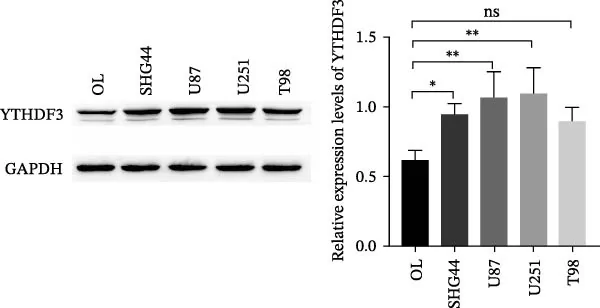
(A)

**Figure:**
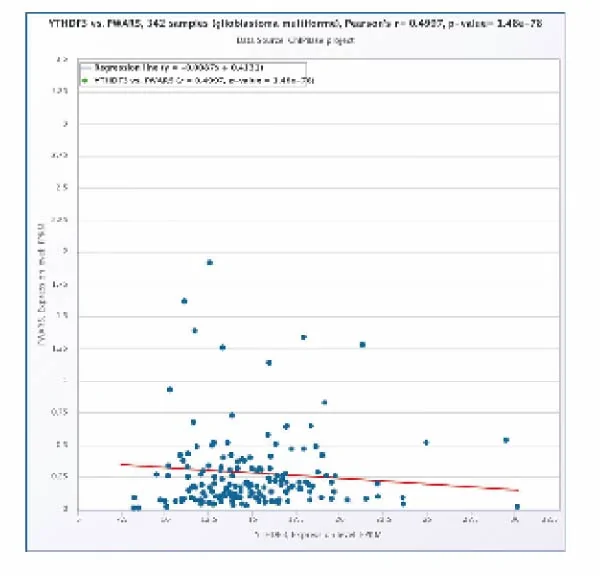
(B)

**Figure:**
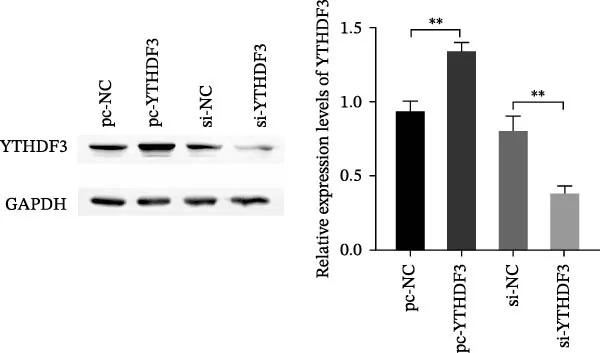
(C)

**Figure:**
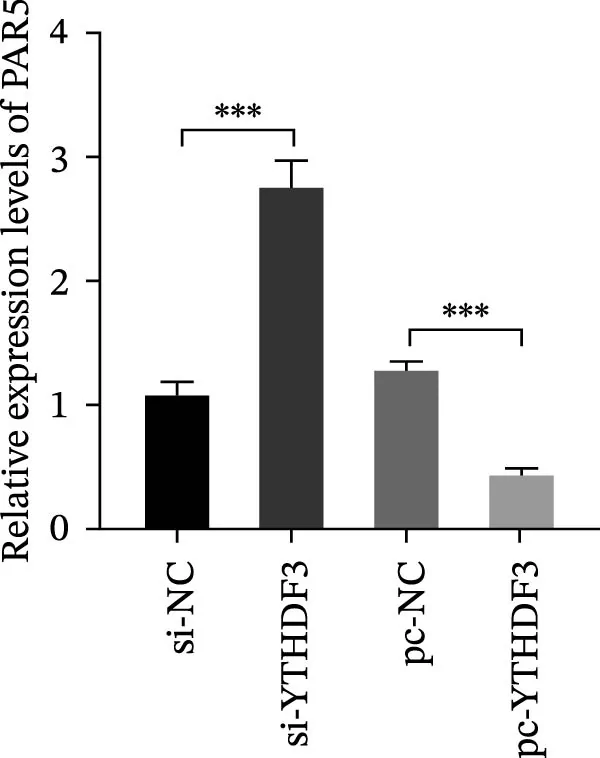
(D)

**Figure:**
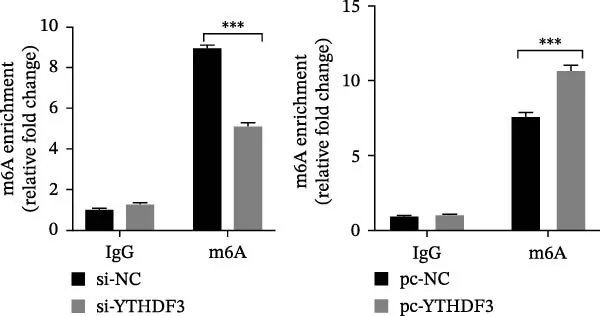
(E)

**Figure:**
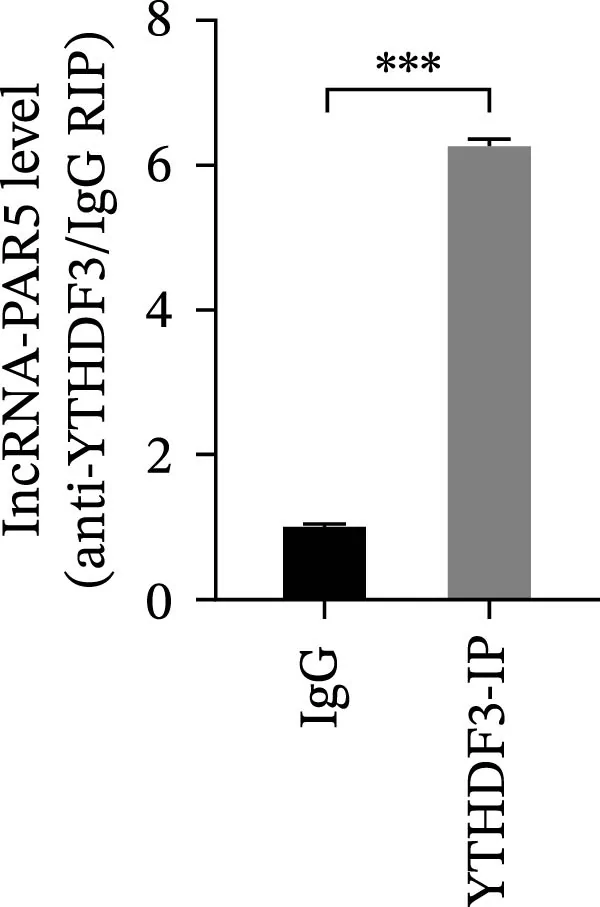
(F)

**Figure:**
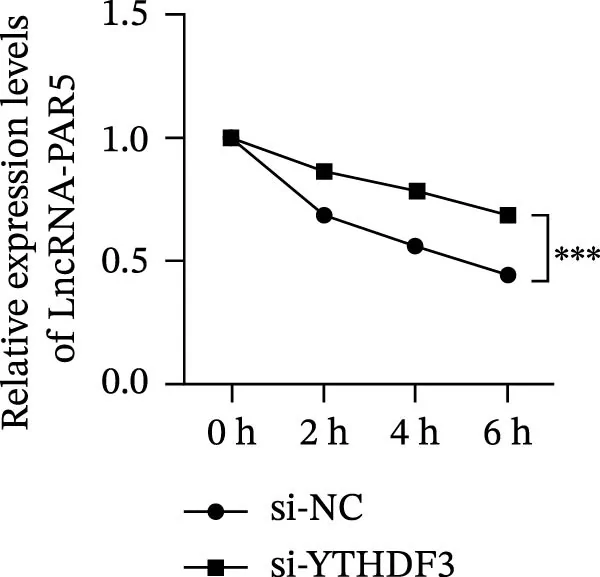
(G)

### 3.4. YTHDF3 Plays a Carcinogenic Role in Glioma Cells

**Figure 4:**
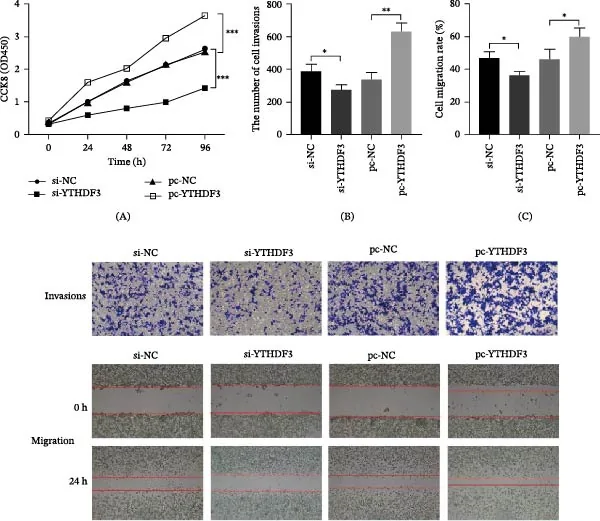
YTHDF3 plays a carcinogenic role in glioma cells. (A) CCK‐8 assay was used to detect cell proliferation activity; (B) Transwell was used to detect cell invasion ability. Scale bar = 50 μm; (C) Wound healing assay was performed to evaluate cell migration ability. Scale bar = 100 μm Compared with NC,  ^∗^ *p*  < 0.05,  ^∗∗^ *p*  < 0.01,  ^∗∗∗^ *p*  < 0.001.

To verify the role of YTHDF3 in glioma cells, cell proliferation, invasion, and migration were detected after knockdown or overexpression of YTHDF3. The results showed that knockdown of YTHDF3 significantly inhibited the proliferation, invasion, and migration of glioma cells, while overexpression of YTHDF3 had the opposite effect (Figure 4A–C). It can be seen that YTHDF3 plays a carcinogenic role in glioma cells.

### 3.5. Rescue of YTHDF3 Knockdown Effects by PAR5 Silencing in Glioma

To clarify whether YTHDF3 promotes glioma progression by regulating PAR5, we systematically examined the effects of knocking down YTHDF3, as well as simultaneously knocking down YTHDF3 and PAR5, on the proliferation, invasion, and migration of glioma cells. The experimental results showed that simultaneous knockdown of YTHDF3 and PAR5 partially reversed the inhibitory effect of YTHDF3 knockdown alone on the malignant behavior of glioma cells (Figure 5A–C). To further validate the biological function of the YTHDF3/PAR5 axis in vivo, we utilized a BALB/c nude mouse subcutaneous xenograft model, implanting U87 cells with stable knockdown of YTHDF3 or simultaneous knockdown of YTHDF3 and PAR5. The experimental results are as follows: in each treatment group, the expression of YTHDF3 (Figure 5F) and lncRNA‑PAR5 (Figure 5G) was effectively downregulated; knockdown of YTHDF3 significantly inhibited tumor growth, as demonstrated by a reduction in tumor volume (Figure 5D) and weight (Figure 5E); simultaneous knockdown of PAR5 partially restored the tumor growth inhibition caused by YTHDF3 deficiency. Notably, in the group with YTHDF3 knockdown alone, the expression of lncRNA‑PAR5 was upregulated, further confirming the negative regulatory role of YTHDF3 on PAR5. Collectively, these results indicate that PAR5 is a key downstream effector molecule through which YTHDF3 drives glioma progression. Inhibition of PAR5 can partially reverse the tumor‐suppressive phenotype induced by YTHDF3 deficiency both in vitro and in vivo.

Figure 5Rescue of YTHDF3 knockdown effects by PAR5 silencing in glioma. (A) CCK‐8 assay was used to detect cell proliferation activity. (B) Transwell was used to detect cell invasion ability. Scale bar = 50 μm. (C) Wound healing assay was performed to evaluate cell migration ability. Scale bar = 100 μm Compared with NC,  ^∗^ *p*  < 0.05,  ^∗∗^ *p*  < 0.01,  ^∗∗∗^ *p*  < 0.001. (D) In vivo xenograft tumor growth model. U87 cells transfected with si‑YTHDF3 or si‑YTHDF3 + si‑PAR5 were subcutaneously inoculated into the axilla of nude mice (*n* = 3 per group). Representative images of xenograft tumors are shown. (E) Quantitative analysis of tumor weights from the xenograft model. (F) Western blot analysis of YTHDF3 expression in xenograft tumors. (G) RT‑qPCR analysis of lncRNA‑PAR5 expression in xenograft tumors. Compared with si‐YTHDF3,  ^∗∗^ *p*  < 0.01,  ^∗∗∗^ *p*  < 0.001.

**Figure:**
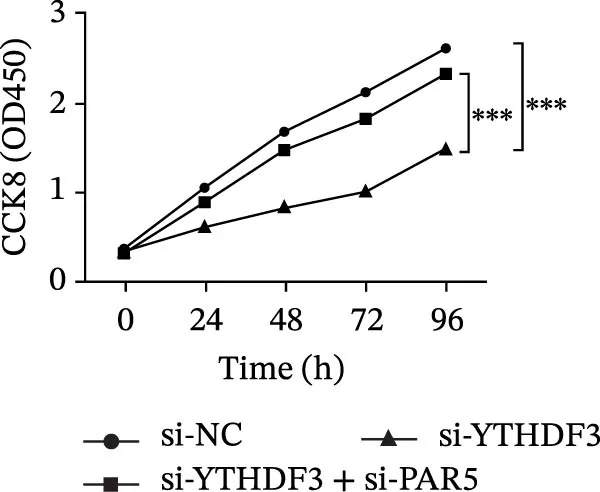
(A)

**Figure:**
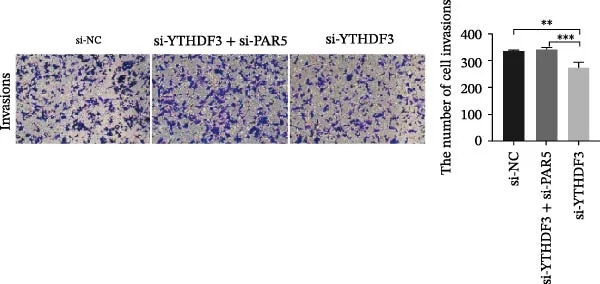
(B)

**Figure:**
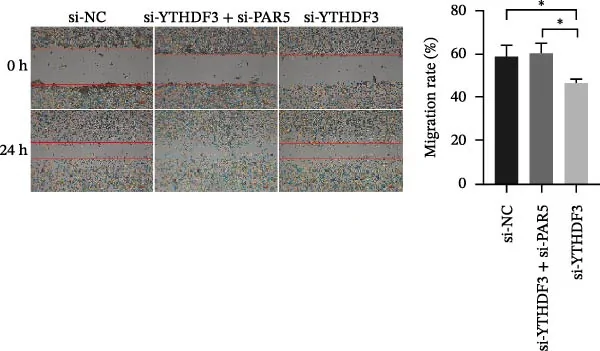
(C)

**Figure:**
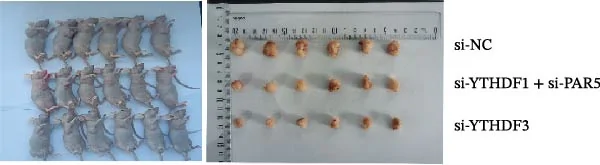
(D)

**Figure:**
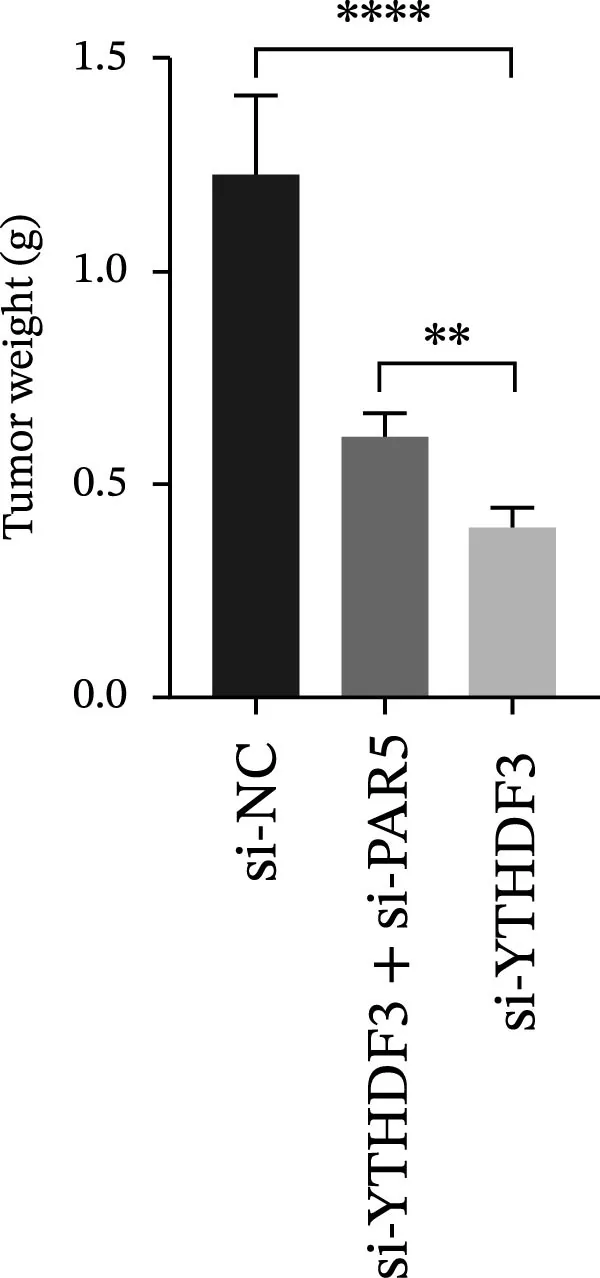
(E)

**Figure:**
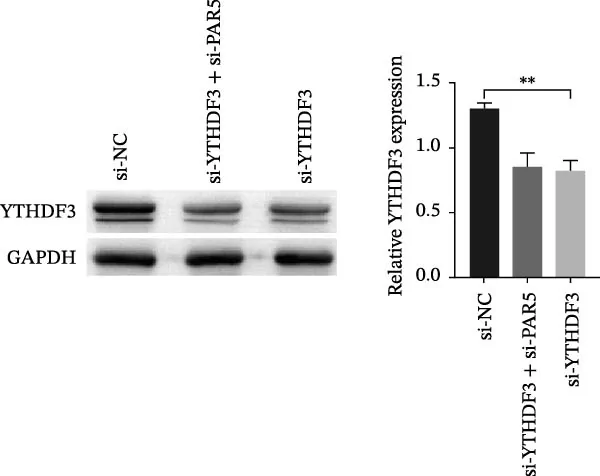
(F)

**Figure:**
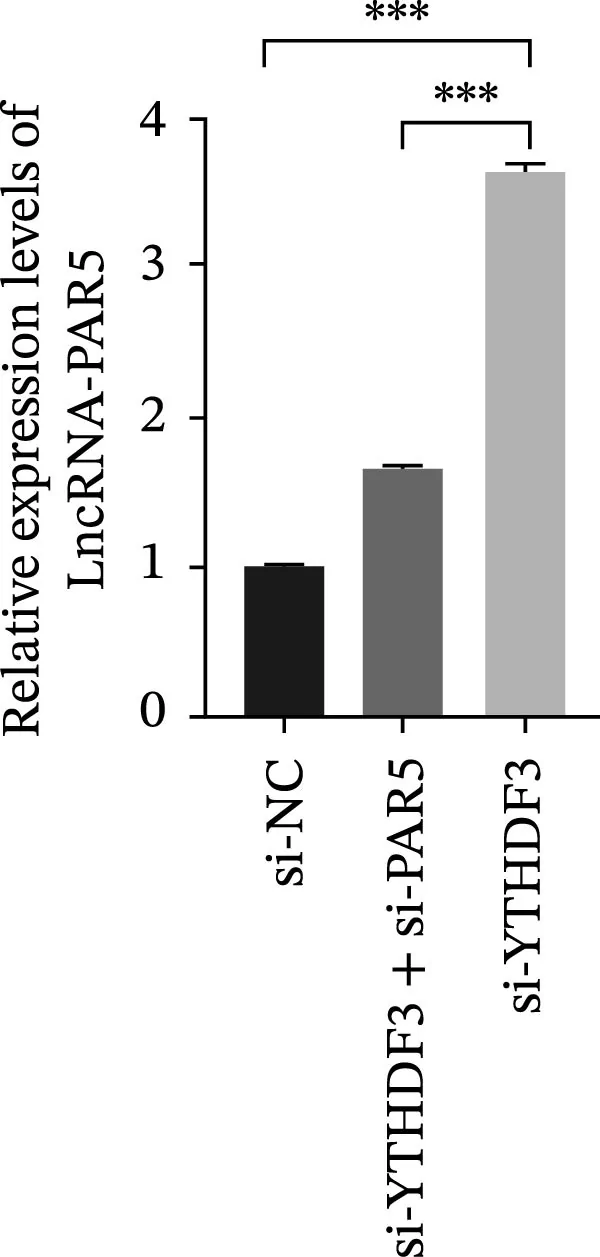
(G)

## 4. Discussion

lncRNA has been widely reported because of its regulatory role in various biological processes of cancer. It is involved in transcription factor activation [30], RNA polyadenylation [31], alternative splicing modification [32], histone modification [33] and gene chromatin rearrangement [34]. In recent years, numerous lncRNAs have been found to play a neuroregulatory role in glioma [35]. For example, lncRNA DLGAP1‐AS2 regulates the development of glioma by up‐regulating YAP1 expression [36]. lncRNA‐ATB promotes TGF‐β‐induced glioma cell invasion through NF‐κB and P38/MAPK pathways [37]. PAR5 has been shown to be involved in the progression of some cancers. For example, Spirito et al. [38] also showed that PAR5 expression was down‐regulated in bladder cancer tissues. Pellecchia et al. [7] confirmed that PAR5 is down‐regulated in anaplastic thyroid cancer and acts as a tumor suppressor by reducing EZH2 activity. Our previous studies have also confirmed that PAR5 can inhibit the proliferation, invasion, and migration of human glioma cells [11], and this study obtained the same results.

A number of studies have shown that m6A regulators are abnormally expressed in glioma and are widely involved in the progression of glioma by targeting lncRNA [39]. For example, METTL3‐mediated m6A‐modified LINC00839 promotes glioma progression and radiation resistance by activating Wnt/β‐catenin signaling [40]. ALKBH5 promotes PD‐L1‐mediated immune escape in glioma through m6A modification of ZDHHC3 [41]. However, it is not clear whether PAR5 expression is regulated by m6A modification in gliomas. We found a potential m6A modification motif in the PAR5 sequence through the SRAMP prediction server, and found that many m6A regulatory genes (METTL14, YTHDF1, YTHDF2, YTHDF3) were abnormally expressed in GBM through GEPIA, indicating that the expression of PAR5 may be regulated by m6A modification. Given the tumor‐suppressive role of PAR5, understanding its regulation by m6A modifications could offer novel insights into glioma biology and potentially unveil new therapeutic targets for intervention.

The fate and function of m6A‐methylated RNA are primarily determined by the reader proteins. As a m6A reader, YTHDF3 can directly bind and read m6A sites on RNA, thus affecting RNA stability, translation, splicing, and output [15, 42]. Several studies have reported that YTHDF3 promotes the translation efficiency of its targets and depends on m6A methylation [15, 43]. YTHDF3 has been shown to contribute to the progression of a variety of tumors, including gastric cancer [44], breast cancer [43], and cervical cancer [18]. A small number of studies have also confirmed that YTHDF3 may be involved in glioma progression. For example, Deng et al. [24] found that YTHDF3 was up‐regulated in glioma tissues; Lee et al. [45] confirmed that YTHDF3 regulates the EGFR/ATK/ERK/p21 signaling axis and promotes cancer progression and osimertinib resistance in glioblastoma cells. However, whether YTHDF3 affects the expression of PAR5 by regulating m6A modification has not been reported. Our study showed that YTHDF3 was up‐regulated in glioma cells, which could bind to PAR5, promote PAR5 m6A modification and inhibit PAR5 expression. This finding not only expands our understanding of YTHDF3’s functional role in glioma but also underscores the potential of targeting m6A regulatory pathways as a therapeutic strategy. Knockdown of YTHDF3 inhibited the proliferation, invasion, and migration of glioma cells, while overexpression of YTHDF3 promoted the proliferation, invasion, and migration of glioma cells. Knockdown of YTHDF3 and PAR5 simultaneously partially restored the inhibitory effect of YTHDF3 knockdown alone on the proliferation, invasion, and migration of glioma cells.

YTHDF3, as a key regulator of RNA stability, YTHDF3 can bind to m6A‐modified lncRNAs, such as DICER1‐AS1, reduce their m6A levels, and accelerate their degradation [46]. In this mechanism, YTHDF3 acts as the “pioneer reader” that initially recognizes nascent m6A‐modified RNAs [47], followed by the coordinated actions of YTHDF1 and YTHDF2: the former promoting translation, and the latter mediating RNA degradation to maintain RNA homeostasis [48]. YTHDF3 and YTHDF2 can recognize m6A‐modified lncRNAs and promote their degradation, thereby weakening their tumor‐suppressive functions and enhancing the malignant properties of cancer cells [42, 49]. In our study, we found that lncRNA‐PAR5 also contains m6A modification sites and is significantly enriched in YTHDF3 immunoprecipitation assays, suggesting that YTHDF3 may bind to PAR5, alter its m6A modification status, and promote its degradation. However, although we have observed the interaction between YTHDF3 and PAR5, along with the enrichment of m6A modifications, whether YTHDF2 is also involved in the degradation of PAR5 still requires further experimental validation.

The regulatory role of m6A modifications in ncRNAs, such as lncRNAs and circRNAs, and their significant value in tumor initiation, progression, and prognostic assessment have become increasingly clear [50]. This field has emerged as a key molecular foundation for constructing clinical prognostic models. For example, in glioma, m6A‐related lncRNAs such as LNCTAM34A have been demonstrated to drive malignant progression by promoting tumor cell proliferation, migration, and the epithelial–mesenchymal transition process [51]. In colorectal cancer, a dual‐marker model based on m6A‐related lncRNAs, including AL135999.1 and AL049840.4, has shown promising prognostic predictive potential [52]. In gastric cancer, the m6A demethylase FTO exerts significant oncogenic effects, likely by regulating the m6A modification of circRNA hsa_circ_0112136, highlighting its potential as a novel diagnostic and therapeutic biomarker [53]. Collectively, these studies underscore the broad potential of m6A‐related lncRNAs in the precision diagnosis and treatment of tumors.

Specifically in glioma, our study demonstrates that the m6A reader protein YTHDF3 promotes glioma cell proliferation, invasion, and migration by suppressing PAR5 expression. Previous research has also indicated that low expression of PAR5 is significantly associated with increased tumor size, higher WHO grade, and reduced Karnofsky Performance Status (KPS), suggesting a strong correlation with poor prognosis in glioma. Survival analysis further confirms that patients with low PAR5 expression exhibit significantly shorter overall survival [11]. However, whether the m6A modification status of PAR5 can serve as a key variable in building a more predictive clinical prognostic model requires further clarification through more systematic mechanistic studies and prospective clinical validation.

In summary, this study reveals the molecular mechanism by which the m6A reader YTHDF3 promotes glioma cell proliferation, invasion, and migration by enhancing PAR5 m6A modification and thereby inhibiting its expression. Targeting the YTHDF3–m6A–PAR5 axis could represent a novel therapeutic strategy for glioma. Modulating m6A reader activity or stabilizing lncRNA‐PAR5 may help restore its tumor‐suppressive function, offering new directions for epigenetic or RNA‐based therapies in the treatment of glioblastoma.

## Author Contributions

Research concept and design: XiangPeng Wang. Collection and/or assembly of data: LiuFei Xu. Data analysis and interpretation: Yuan Xu, YongBin Duan, Bo Wang, YiYu Luo. Writing the article: LiuFei Xu. Critical revision of the article: XiangPeng Wang.

## Funding

This study was supported by the Science and Technology Plan Project of the Department of Science and Technology of Yunnan Province (Basic Research, Grant No. 202301AY070001‐162), the Yunnan Fundamental Research Projects at Kunming Medical University (Grant No. 02301AY070001‐162, Project Title: “Study on the mechanism of lncRNA‐PAR5 inhibiting EZH2‐mediated IAK2/STAT3 pathway activation to reduce malignant progression of glioma”), and the Research Project of Yunnan Clinical Medical Center for Neurocardiac Diseases (Grant No. 2024YNLCYXZX0058).

## Disclosure

All authors read and approved the final manuscript.

## Ethics Statement

The Animal Experiment Ethics Review Committee of Kunming Medical University (kmmu20230611). This study was carried out in strict accordance with the recommendations in the Guide for the Care and Use of Laboratory Animals of the National Institutes of Health. All methods are reported following ARRIVE guidelines (https://arriftguidelines.org) for the reporting of animal experiments.

## Consent

The authors have nothing to report.

## Conflicts of Interest

The authors declare no conflicts of interest.

## Data Availability

Data available on request from the authors.
